# The effect of the structure of a helical nanofilament of the B4 phase of bent-core liquid crystals on the nano-phase separation mixed with a rod-like cholesteric liquid crystal mixture

**DOI:** 10.1039/d2ra03316j

**Published:** 2022-10-13

**Authors:** Yoichi Takanishi, Fumito Araoka, Hiroshi Iwayama

**Affiliations:** Department of Physics, Kyoto University Kitashirakawaoiwake-cho, Sakyo-ku Kyoto 606-8502 Japan ytakanis@scphys.kyoto-u.ac.jp; RIKEN Center for Emergent Matter Science (CEMS) 2-1 Hirosawa Wako Saitama 351-0198 Japan; UVSOR Synchrotron Facility, Institute for Molecular Science Okazaki Aichi 444-8585 Japan; School of Physical Sciences, The Graduate University for Advanced Studies (SOKENDAI) Okazaki 444-8585 Aichi Japan

## Abstract

We studied the structure of a helical nano-filament of the B4 phase in mixtures of a cholesteric liquid crystal mixture and a bent-core molecule using a resonant soft X-ray scattering (RSoXS) technique. In this system, nanophase separation occurs and it was already found that an unexpected new functional chiral smectic structure in the rod-like molecule rich region is constructed by the strong interaction between bent-core and rod-like molecules. In this paper, we focused on the structure of the helical filament in the bent-core liquid crystalline molecule rich region in this mixing system, and it was found that the pitch of the helical filament decreases and the coherence of the helical structure increases.

## Abstract 2

We studied the structure of a helical nano-filament of the B4 phase in mixtures of a cholesteric liquid crystal mixture and a bent-core molecule using a resonant soft X-ray scattering (RSoXS) technique.

## Graphical abstract

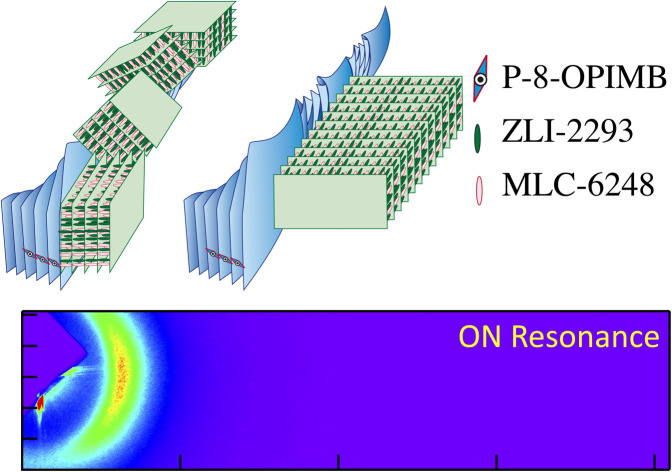

## Introduction

Due to specific intermolecular interactions, frustration and competition, soft matter is expected to have various new structures and functionality. Liquid crystals are one such material. For example, various Sm-C like phases are observed by the competition of ferroelectric and antiferroelectric phases.^1^ The antiphase structure appears due to the hydrogen bonding between interlayers.^2^ By strong geometrical frustration between nematic ordering and the surface anchoring, stable topological defects appear.^3^ Roberts *et al.* reported that combining structural elements promoting the formation of SmA and SmC phases in the same molecules causes a frustration that enhances de Vries-like properties.^4^ Bent-core mesogenic molecules show specific liquid crystalline phases, called B1–B8 and SmAP, different from the mesophases formed by rod-like mesogenic molecules, because of the specific intermolecular interactions caused by its molecular shape and molecular packing in a smectic layer.^5^ Among them, the B4 phase exhibits spontaneous chiral separated domains under polarized microscopy, and it forms a helical structure with a pitch of several hundred nanometers by several twisted smectic layers due to the layer chirality.^6^

**Fig. 1:**
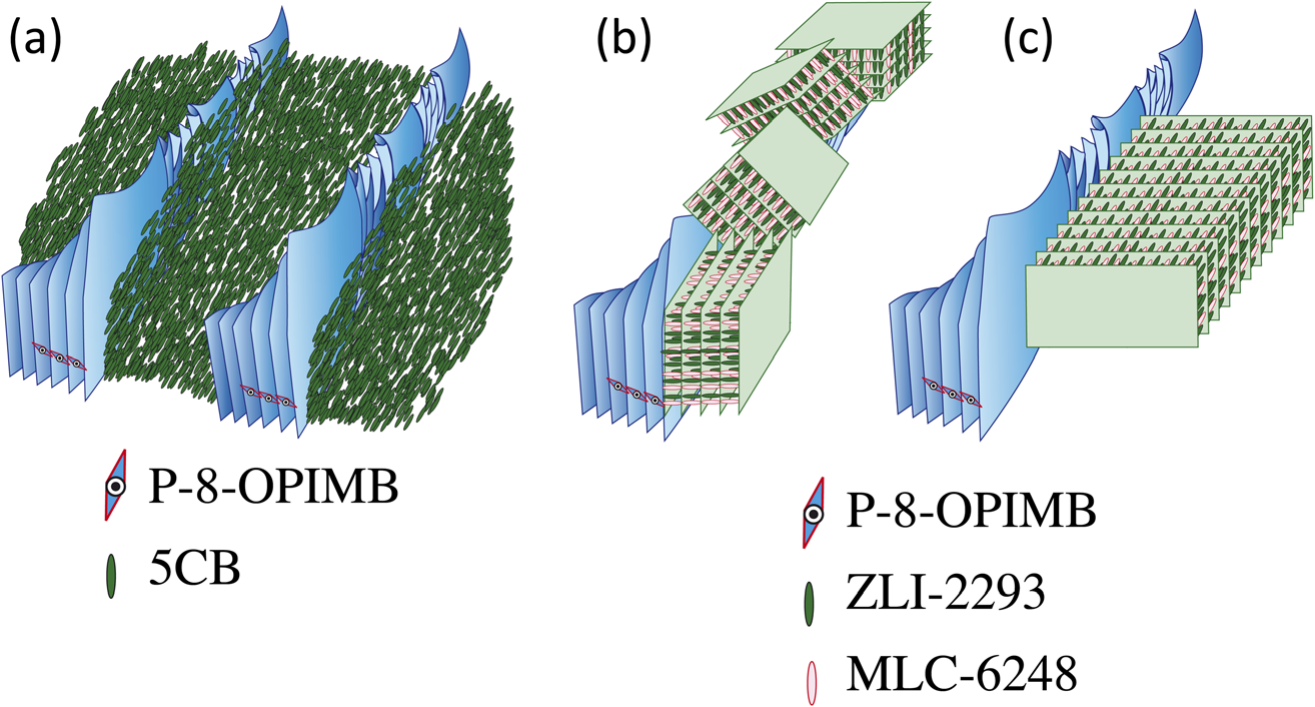
(a) Schematic image of nanophase separation of the binary system of bent-core and rod-like nematic molecules.^8^ (b) and (c) Schematic images of two types of nanophase separation of the binary system of a bent-core molecule and rod-like cholesteric liquid crystal mixture.^10^

One of authors (YT) and colleagues studied the binary system of rod-like (pentyl-cyano biphenyl, 5CB) and bent-core molecules (1,3-phenylene bis[4-(4-8- alkoxyphenyliminomethyl)-benzoates], P-8-OPIMB),^7–9^ and found the nano-scale phase separation of the nematic domain was dominated by 5CB and the helical nanofilament dominated by P-8-OPIMB, as shown in Fig. 1(a). Very recently, YT studied the binary system composed of a rod-like cholesteric liquid crystal (CLC) mixture and a bent-type liquid crystal showing a B4 phase^10^ to confirm whether the nano-scale phase separation as observed in the 5CB–P-8-OPIMB binary system is formed in this system or not. As a result, YT unexpectedly discovered a new smectic structure with wide temperature range that does not appear in unmixed constituent molecules, in addition to confirmation of the nano-scale phase separation, as shown in Fig. 1(b) and (c).^10^ This newly emerged smectic structure seems to be fundamentally composed of a rich concentration of rod-like molecules and its stabilization seems to be caused by strong interaction with bent-core molecules.

On the other hand, in this system, there remains an open question with respect to the effect of the nano-helical filament of the B4 phase^6^ consisting of the bent-core liquid crystal molecules. In this paper, we report the local nano-structure analysis of B4 nano-helical filament on the nano-phase separation mixed with rod-like cholesteric liquid crystal mixture using resonant soft-X-ray scattering (RSoXS) technique.^11–13^ Concentration dependence of rod-like molecules and temperature dependence were measured, and the mixing effect on the helicity and correlation of B4 nano-helical filament are discussed.

## Experimental

**Fig. 2:**
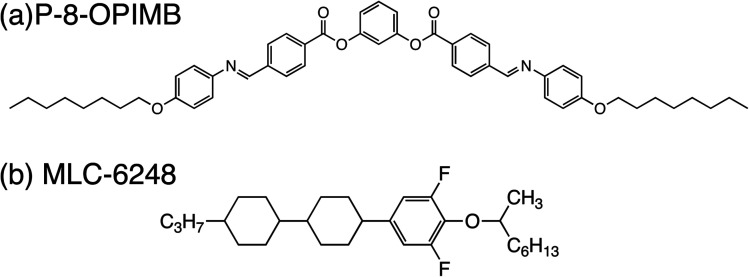
Chemical structures of (a) P-8-OPIMB (compound B) and (b) MLC-6248 (compound C).

Used achiral bent-core liquid crystal was 1,3-phenylene bis[4-(4-8-alkoxyphenyliminomethyl)-benzoates] (P-8-OPIMB, Iso 173.9 °C B2 152 °C B3 140 °C B4 (ref. 5–8)) and a rod-like CLC mixture was composed of a nematic mixture (ZLI-2293, Merck) doped with a chiral rod molecule (MLC-6248, Merck). Chemical structures of MLC-6248 and P-8-OPIMB are shown in Fig. 2. In this paper, ZLI-2293 (Iso 78 °C N > 0 °C), P-8-OPIMB and MLC-6248 (Iso 74 °C Ch 44 °C Sm) are referred to A, B and C, respectively. Mostly used mixing ratio of cholesteric liquid crystal mixture (mixture AC) is A : C = 75 : 25. In this mixture, the phase sequence is Iso 78 °C Ch > 0 °C. From the previous paper,^10^ it was found that these mixtures show nano-scale phase separation of bent-core LC-rich B4 helical filament and a new smectic structure dominated by rod-like molecules, and the new smectic structure was stabilized until 90 °C.

For the RSoXS measurement, sample was sandwiched by two 100 nm-thick silicon-nitride membranes (NORCADA or NTT-AT), and prepared from the isotropic phase (140–160 °C, depending on the mixture concentration) to room temperature by gradual cooling process (0.1 °C min^−1^). Sample thickness was about several hundreds of nanometers, judging from the color of the optical birefringence.

**Fig. 3:**
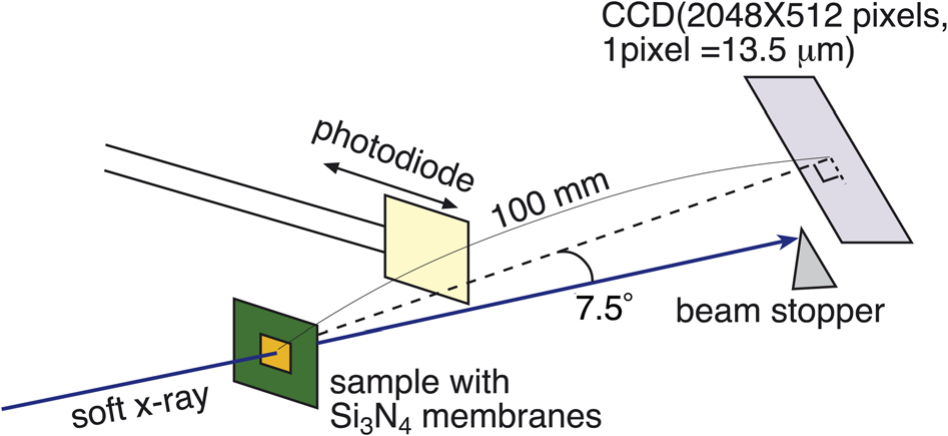
Experimental setup of BL3U for small angle SoXRS measurement.

**Fig. 4:**
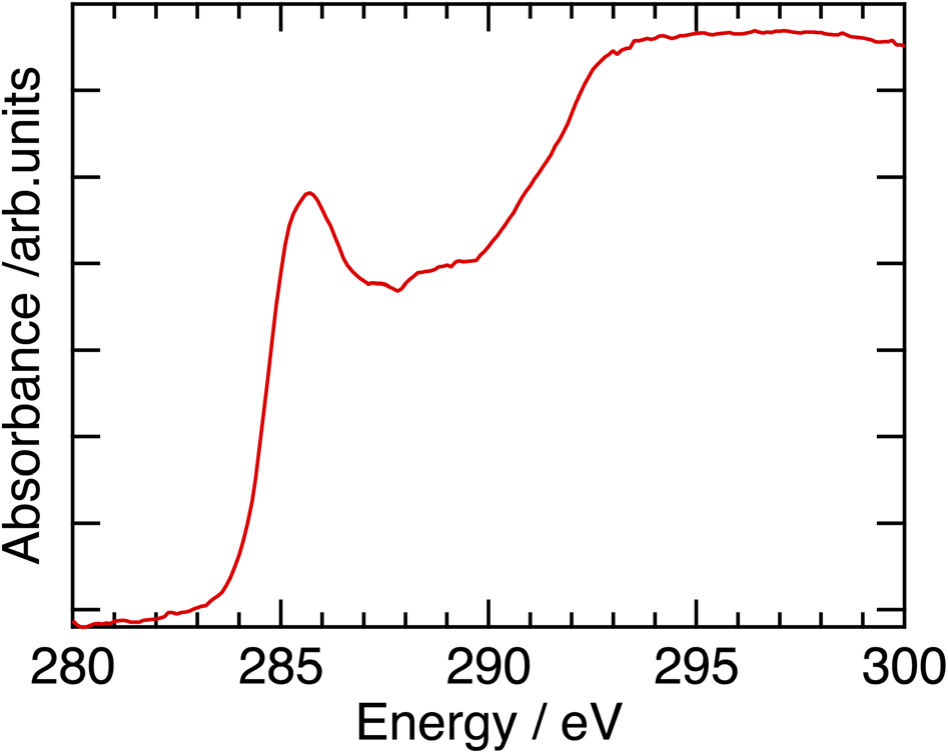
Absorption spectrum around soft X-ray region of pure P-8-OPIMB at room temperature (B4 phase).

RSoXS measurements were performed at BL3U of UVSOR beamline of Institute for Molecular Science (Okazaki, Aichi). Experimental setup was depicted in Fig. 3. Sample was placed in the vacuum chamber, and the degree of vacuum around the sample was kept to be less than 10^−2^ Pa. (The degree of vacuum of X-ray beam introduction part was *ca.* 1 × 10^−6^ Pa.) Temperature of samples was controlled by hand-made oven with temperature controller (DB-1230, CHINO). The scattering was detected by CCD camera (DO940P-BN, ANDOR), and camera length was 100 mm, and the CCD camera can cover the scattering angle from about −0.4 deg to 15.3 deg. Incident X-ray energy was tuned from 270–290 eV, which can cover the absorption energy of carbon K-edge (*ca.* 285 eV). Behind the sample, the photodiode for measuring the transmittance of incident X-ray beam can be inserted and removed. Typical absorption spectrum was shown in Fig. 4, and carbon K-edge energy in this compound was 285 eV. By fitting the X-ray profiles using the following Lorentzian equation, half of helical pitch and the correlation length were determined.1
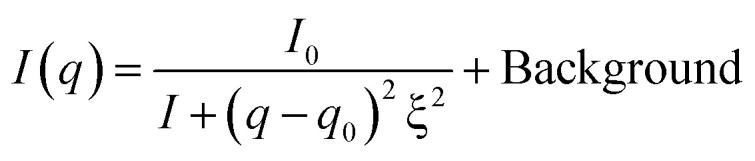


## Results and discussion

**Fig. 5:**
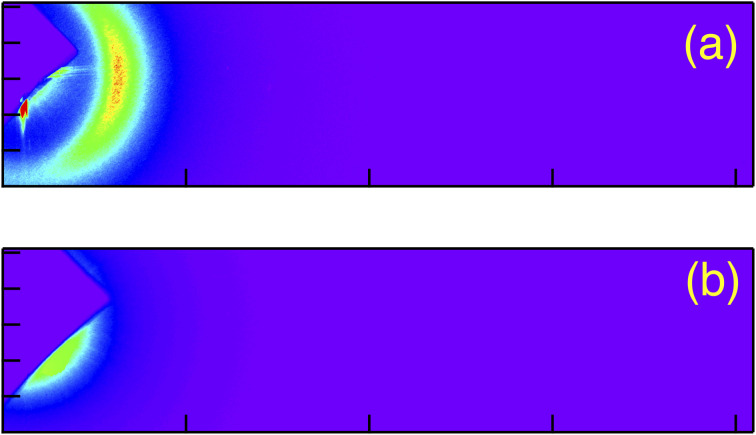
2D scattering image of pure P-8-OPIMB (compound B) at incident X-ray energy equal to 285.5 eV(on resonance) (a) and 275 eV(off resonance) (b).

**Fig. 6:**
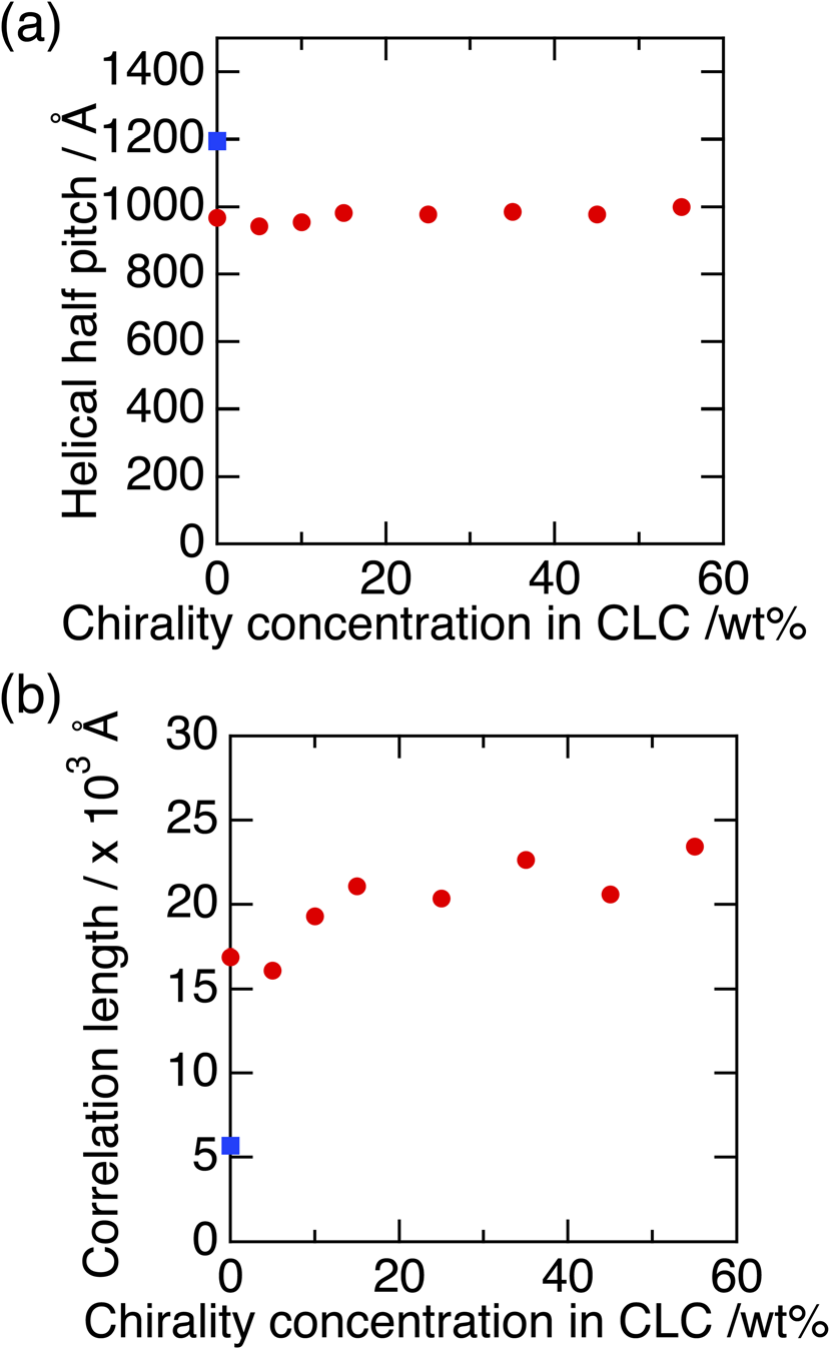
Half pitch of helical nano-filament obtained from small angle RSoXS peak (a) and its correlation length (b) as a function of chiral dopant concentration. Blue square symbols show the data obtained from pure P-8-OPIMB (compound B).

Fig. 5 shows the typical obtained 2 dimensional RSoXS images of pure P-8-OPIMB when the incident X-ray energy was (a) 285 eV (on resonance) and (b) 275 eV (off resonance). In the on-resonance condition, a clear sharp peak was observed, as reported by Zhu *et al.*,^12,13^ which corresponds to the half pitch of nano-scale helical structure (about 1200 Å). Fig. 6(a) and (b) show the half pitch and its correlation length as a function of chiral dopant (C) concentration. Here, the mixing ratio of B and mixture AC was fixed at B : AC = 3 : 7, but the mixing ratio of A and C changes. The helical pitch of the mixture ABC was about 960 Å, which is shorter than that of pure P-8-OPIMB (compound B, shown by blue square symbol), and the magnitude of the half pitch of the mixture ABC is independent of chiral dopant (C) concentration. In general, helicity was influenced by the amount of the chiral dopant; with the increases in the concentration of chiral dopant, the twisting power of the system increases and the helical pitch becomes shorter. However, this result indicates that the pitch is independent of the concentration of chiral dopant, which suggests the chiral molecules (compound C) hardly influence the twisting power of the nano-helical filament of the B4 phase.

On the other hand, the correlation length of mixtures is about three times longer than that of pure P-8OPIMB (blue symbol), and it shows the chiral dopant concentration dependence; the correlation length tends to increase slightly as the chiral dopant increases, suggesting that the helical structure of nano filament in bent-core molecules rich region is spatially homogeneous compared to the pure compound B. This is because when the nano-filaments grow in the isotropic states of rod-like molecules (A and C) rich region, the filaments are less likely to hinder each other to grow freely.

**Fig. 7:**
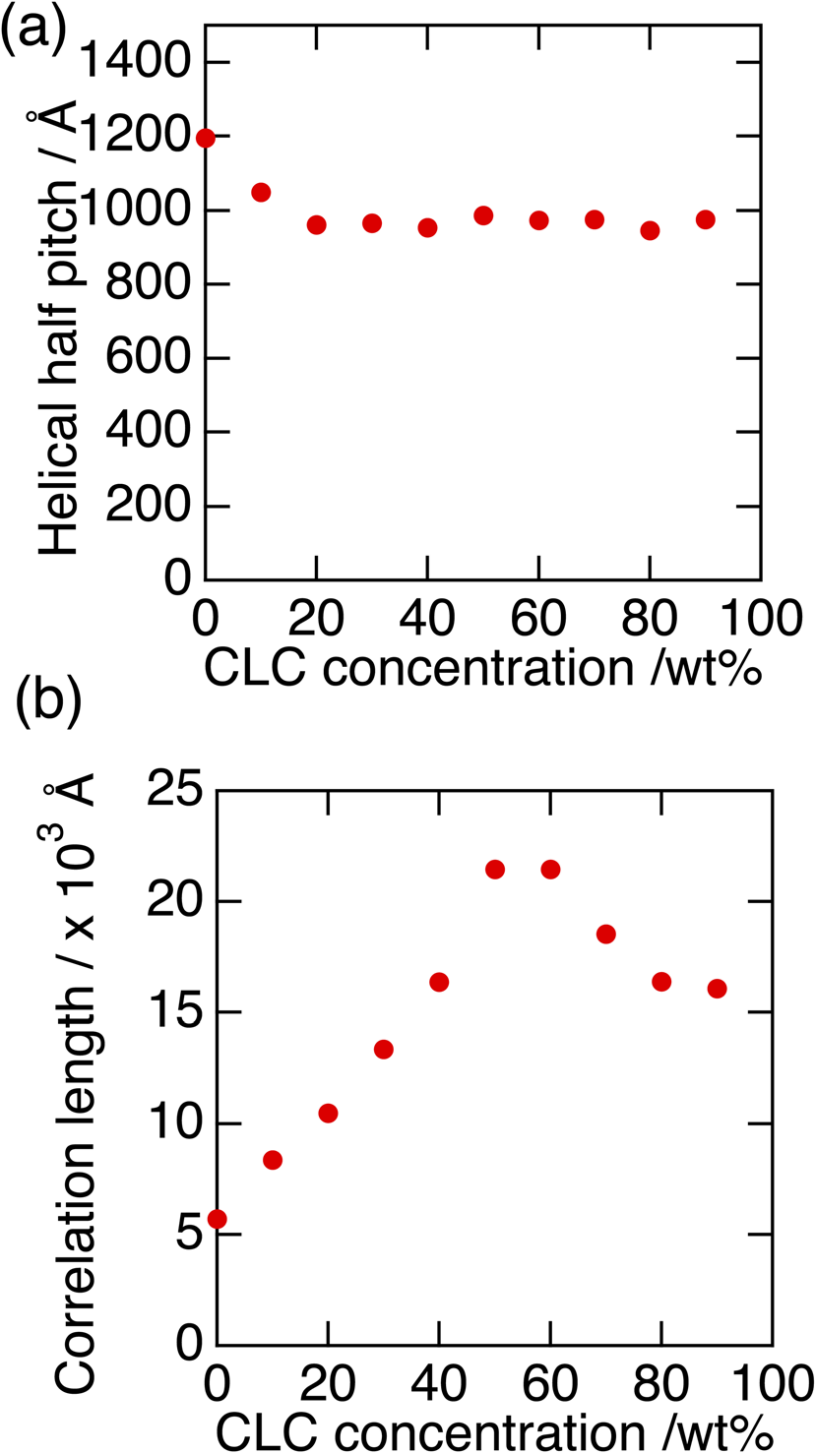
Half pitch of helical nano-filament obtained from small angle RSoXS peak (a) and its correlation length (b) as a function of the concentration of mixture AC.

Fig. 7(a) and (b) show the half pitch and its correlation length as a function of mixture AC concentration. Here, the mixing ratio of compounds A and C was fixed at A : C = 7 : 3. The helical pitch becomes shorter when the concentration of the mixture AC is up to 20 wt%, and there hardly changes at higher concentration than 20 wt%. On the other hand, the correlation length increases with increase in the concentration of the mixture AC until 60 wt%, and the it becomes four times as long as that in the pure compound B. This increase would also come from the same origin of results in Fig. 6(b); with increase in the concentration of mixture AC, each filament is considered to grows more freely without hindering. In case of more than 70 wt% mixture, the correlation length slightly decreases, because the fitting deviation by Lorentzian equation might seem to be larger due to the low scattering intensity.

## Summary

As a summary, the structure of helical nano-filament of B4 phase in the nano phase separation of a cholesteric liquid crystal mixture and a bent-core molecule was analyzed using resonant soft X-ray scattering (RSoXS) techniques. In the previous paper, it was reported that an unexpected new functional chiral smectic structure in rod-like molecule rich region is constructed by the strong interaction between bent-core and rod-like molecules, in spite of the nanophase separation. Even in the helical nano-filament of B4 phase of the bent-core liquid crystalline molecules, it was found that the pitch of helical filament decreases and coherence of helical structure increases, which would be caused by the interaction between bent-core and rod-like molecules.

## Conflicts of interest

There are no conflicts to declare.

## Supplementary Material
